# Photoelectrochemical Performance Observed in Mn-Doped BiFeO_3_ Heterostructured Thin Films

**DOI:** 10.3390/nano6110215

**Published:** 2016-11-16

**Authors:** Hao-Min Xu, Huanchun Wang, Ji Shi, Yuanhua Lin, Cewen Nan

**Affiliations:** 1 State Key Laboratory of New Ceramics and Fine Processing, School of Materials Science and Engineering, Tsinghua University, Beijing 100084, China; xuhm13@mails.tsinghua.edu.cn (H.-M.X.); wanghc12@mails.tsinghua.edu.cn (H.W.); cwnan@mail.tsinghua.edu.cn (C.N.); 2 High-Tech Institute of Xi’an, Xi’an 780025, China; 3 Department of Metallurgy and Ceramics Science, Tokyo Institute of Technology, 2-12-1, Ookayama, Meguro-ku, Tokyo 152-8552, Japan; shi.j.aa@m.titech.ac.jp

**Keywords:** photocatalysis, photoelectrochemical, solar energy conversion, Mn-doped BiFeO_3_ films, heterostructure

## Abstract

Pure BiFeO_3_ and heterostructured BiFeO_3_/BiFe_0.95_Mn_0.05_O_3_ (5% Mn-doped BiFeO_3_) thin films have been prepared by a chemical deposition method. The band structures and photosensitive properties of these films have been investigated elaborately. Pure BiFeO_3_ films showed stable and strong response to photo illumination (open circuit potential kept −0.18 V, short circuit photocurrent density was −0.023 mA·cm^−2^). By Mn doping, the energy band positions shifted, resulting in a smaller band gap of BiFe_0.95_Mn_0.05_O_3_ layer and an internal field being built in the BiFeO_3_/BiFe_0.95_Mn_0.05_O_3_ interface. BiFeO_3_/BiFe_0.95_Mn_0.05_O_3_ and BiFe_0.95_Mn_0.05_O_3_ thin films demonstrated poor photo activity compared with pure BiFeO_3_ films, which can be explained by the fact that Mn doping brought in a large amount of defects in the BiFe_0.95_Mn_0.05_O_3_ layers, causing higher carrier combination and correspondingly suppressing the photo response, and this negative influence was more considerable than the positive effects provided by the band modulation.

## 1. Introduction

BiFeO_3_ (BFO) is famous for its room temperature multiferroic properties, but it is attracting more and more interest as a small band gap photo-ferroelectric material. In the past few years, photocatalytic (PC), and photovoltaic (PV) properties of BFO films have been studied [1,2,3,4,5]. For example, polycrystalline BFO photo electrode was applied for water photo-oxidation [6]; an enhanced ferroelectric PV response of multilayered BiFeO_3_/BaTiO_3_ was observed [7]. The photoelectrochemical (PEC) response of BFO with ferroelectric materials has also been reported [8,9]. Findings imply the promising potential applications of BFO films in terms of photocatalysts, photovoltaic cells, multifunction sensors, etc. [10,11,12].

High quality photocatalyts, or the PEC electrodes, are believed to possess high-energy conversion efficiency. One of the key issues for energy conversion performance is the ability of light absorption, which is mostly decided by the band gap of the material. Element doping is quite a normal method in band engineering. Usually, metal element doping does better in narrowing the band gap than the anions do [13]. More specifically, to narrow the band gap of BFO, people can conduct A/B-site doping with elements such as La [14], Gd [15], Y and Ti [16]. The A-site and B-site represents the positions of Bi and Fe atoms in the BFO lattice, respectively. B-site Mn doping is a potential method of modifying the band structure of BFO [17]. The Mn element has attracted great attention due to its special role in BFO thin films. For example, people found that BFO films showed enhanced ferroelectric and weak magnetic behaviors by 5% Mn substitution [18,19]. Optical and PC properties of Mn-involved co-doping BFO have also been recently studied. For example, researchers observed that Ba/Ca and Mn co-doped BFO nanofibers delivered enhanced PC performances [20,21]; Vinay et al. obtained a photo-induced current of about 250-fold in Ce and Mn co-doped BFO thin films at zero bias in a PV test [5]. However, few studies have focused on the effects of the Mn single doping of BFO on PEC properties. In this work, we set Mn doping content as 0.05, preparing BiFe_0.95_Mn_0.05_O_3_ thin films, and studied their optical and PEC properties.

Furthermore, the energy conversion is highly restricted by the combination of photo-induced electro-hole pairs, which can happen inside or on the surface of the material. How to separate these pairs much more efficiently has been a question for a long time [22]. According to the research before, we know that the internal field plays an important role in separating e–h pairs; through studying the principles of the internal field, we are able to create or design an internal field that could provide more effective electron-hole separation, faster interface charge transfer, and longer carrier lifetime. For example, using the polarization in the ferroelectric materials [9,23,24], constructing the p-n junction or polymorph junction can work appropriately [25]. Considering these facts, it would be interesting to construct a heterostructure with BFO and band-modified BFO, and study the effects of internal field built around boundary on PEC activity. Therefore, in the present work, we made heterostructured BFO/BiFe_0.95_Mn_0.05_O_3_ (BFMO) films and investigated their PEC properties.

## 2. Results and Discussion

**Figure 1:**
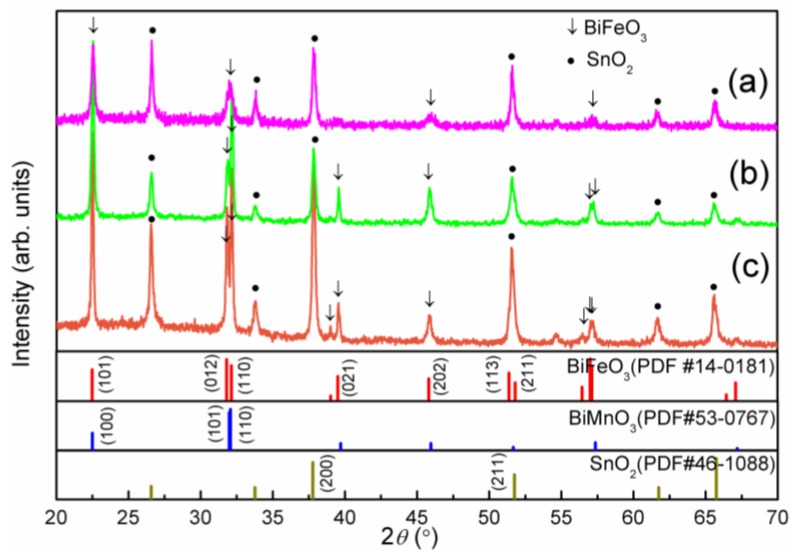
X-ray diffraction (XRD) patterns of (**a**) BiFe_0.95_Mn_0.05_O_3_ (BFMO); (**b**) BiFeO_3_ (BFO)/BFMO and (**c**) BFO films. PDF#14-0181 shows BFO in rhombohedral structure, and PDF#53-0767 shows BFMO in tetragonal structure, PDF#46-1088 shows the peak positions of SnO_2_ substrate.

**Figure 2:**
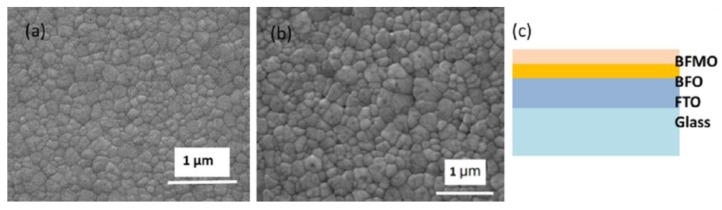
The surface scanning electron microscopy images of (**a**) BFO and (**b**) BFMO film; (**c**) The illustration of BFO/BFMO heterostructure.

X-ray diffraction (XRD) results are shown in Figure 1. All peaks in the XRD patterns of the BFO films are indexed for a rhombohedral structure with R3m symmetry and were found to be in good agreement with the corresponding values reported in the PDF#14-0181, while the BFMO films display a structure transition according to the (012) and (110) peaks that were changed, and the (021) peak with the three peaks around 2θ = 56°–57.1° disappeared. The peaks of the BFMO films fit well with the tetragonal BiMnO_3_ reported in PDF#53-0767, which implies that the film structure has partly transferred from rhombohedral to tetragonal due to the Mn doping. Similar results have been reported previously [18]. From the XRD patterns, we can also see that BFMO did not crystalize as well as BFO. Both the BFO film and the BFMO film is densely crystalized according to the scanning electron microscopy (SEM) images of the surface morphology of the films (Figure 2a,b).

**Figure 3:**
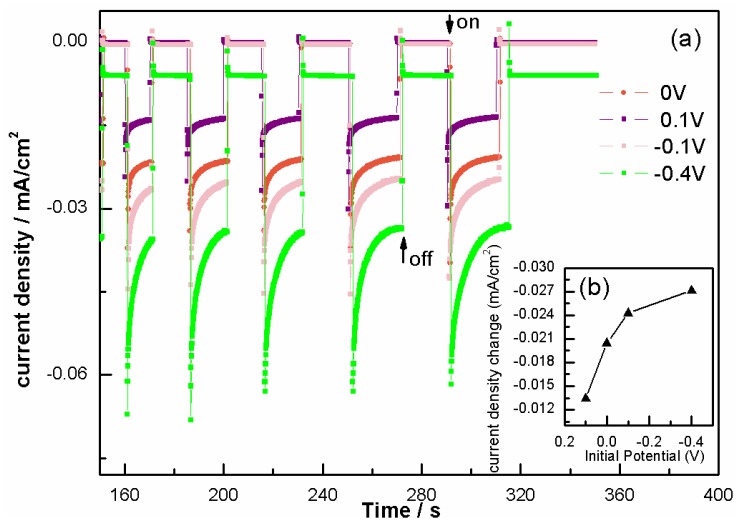
(**a**) *I-t* curves of BFO at 0 V, 0.1 V, −0.1 V and −0.4 V bias voltages; inset (**b**) is the photocurrent density change under different bias voltages.

**Figure 4:**
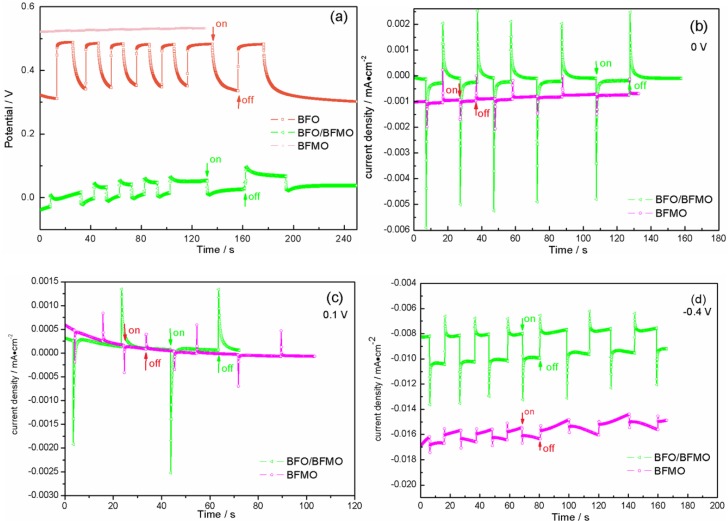
(**a**) Open circuit potential tests of the three types of samples under on-off 150 mW/cm^2^ light illumination. *I*-*t* curves at (**b**) 0 V; (**c**) 0.1 V; (**d**) −0.4 V of BFMO and BFO/BFMO films.

To test the photo-response ability of our films, we first tested the PEC properties of BFO. According to the *I*-*t* measurements (Figure 3, 0 V bias) and *P*-*t* (Figure 4a, BFO), we observed a repeatable and stable instantaneous response to the light illumination, coming along with a photocurrent density −0.023 mA·cm^−2^ and an open circuit potential (OCP) −0.18 V when exposed to light, proving again the high sensitivity of BFO films to light. The −0.023 mA·cm^−2^ photocurrent density at zero bias voltage was much larger than previous reports [8,26]. The *I*-*t* test with the varied initial potentials of BFO (Figure 3) implies that a negative initial potential can improve the photo response, while a positive one will weaken the response, which can be explained by the fact that negative OCP appears under photo illumination, and the photo-induced electrons aggregating on the F-doped SnO_2_ (FTO) side have to get over the barrier that the positive applied voltage creates to move out to the counter electrode. The dark current density increased to −0.005 mA·cm^−2^ at an applied potential of −0.4 V, which is much smaller than previous reports [26].

Then, we tested the photo-response ability of BFMO films and the heterostructured BFO/BFMO films and compared them with BFO films. BFMO displayed a nearly 0 V OCP, and BFO/BFMO appeared to be unstable, with only a quarter OCP to BFO (Figure 4a). In Figure 4a, the initial position of the three types varied because of the different surface state contrast to the reference electrode. The time dependence of the photocurrent density measurements at 0 V, 0.1 V and −0.4 V bias voltages are shown in Figure 4b–d. BFMO films and the BFO/BFMO films barely had an obvious photocurrent. The values are still small, even when applying a negative voltage. Even though Mn doping in BFO will always induce a low leakage current (the dark current intensity of BFMO at 0 V is 8 × 10^−4^ mA·cm^−2^, slightly larger than BFO of 2.5 × 10^−4^ mA·cm^−2^), it cannot affect the photocurrent that much, since the dark current intensity of BFO/BFMO is 9 × 10^−5^ mA·cm^−2^, much smaller than BFO. According to the *I*-*t* results of BFMO and BFO/BFMO films, the transient anode photocurrent upon light illumination was small and attenuated quickly, indicating the strong recombination and short life of photo-induced carriers. In general, pure BFO film performs best compared with BFMO and the heterostructured BFO/BFMO films.

**Figure 5:**
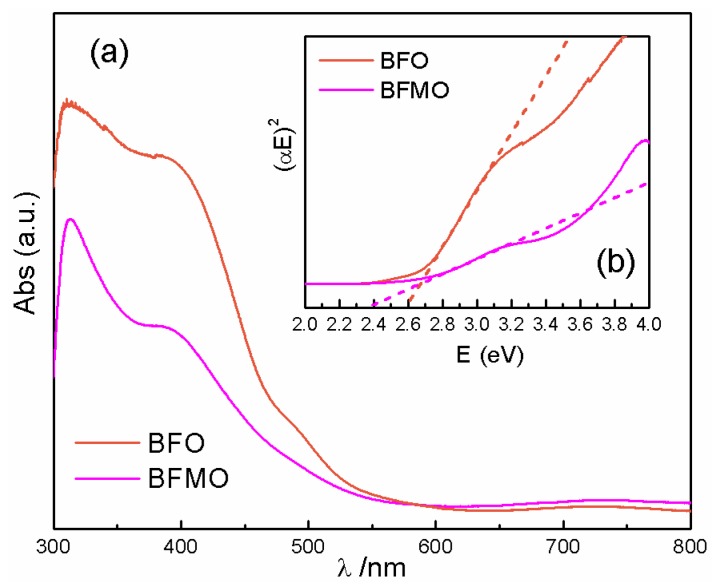
(**a**) Ultraviolet-visible (UV-Vis) absorption spectra of BFO and BFMO; (**b**) the band gap calculation.

**Figure 6:**
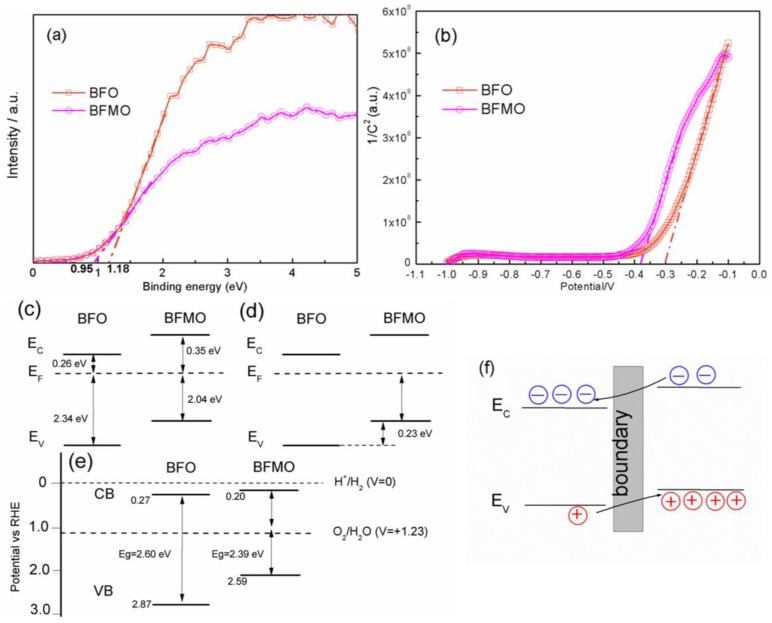
(**a**) X-ray photoelectron spectroscopy (XPS) valance spectra; (**b**) Mott–Schottky curve; the band position determined by (**c**) theoretical calculation, (**d**) XPS valance spectra method and (**e**) Mott–Schottky method; (**f**) an illustration of carriers transferring through boundary.

To explain the phenomenon above, the energy band structures of the films were carefully investigated. Ultraviolet-visible (UV-Vis) absorption measurements and the band gap calculations (shown in Figure 5) indicate that BFMO cannot absorb light as much as BFO does, and it has a band gap of 2.39 eV, which is smaller than BFO’s band gap of 2.60 eV. To get an insight into how the band structure of BFO changes via Mn doping, and to understand the internal field built around the boundary of heterostructured BFO/BFMO, several methods were applied to determine the band position of BFO and BFMO in the heterostructured BFO/BFMO. The first method relies on the following formula:

(1) $$E_{C}=X-E_{e}-\frac{1}{2}E_{g}$$

where *E_C_* presents the position of conductive band (CB), *X* is the absolute electronegativity of the semiconductor, *E_e_* is the energy of free electrons on the hydrogen scale, and *E_g_* is the band gap of the semiconductor. The theoretical band positions are shown in Figure 6c. The second method is represented by using X-ray photoelectron spectroscopy (XPS) valance spectra in Figure 6a, which can explain *E_v_*, the position of valence band (VB) in Figure 6d, but it is not accurate enough [27]. The third method is the Mott–Schottky method, which is conducted by the electrochemical station. The Mott–Schottky equation is as follows:

(2) $$\frac{1}{C^{2}}=\frac{2}{{\varepsilon \varepsilon }_{0}A^{2}eN_{D}}(V-V_{fb}-\frac{k_{B}T}{e})$$

where *C* is space charge layers capacitance, e is electron charge, ε is dielectric constant, ε_0_ means permittivity of vacuum, *N_D_* = electron donordensity, *V* means applied potential, *k_B_* is Boltzmann’s constant, *T* is absolute temperature, and *V_fb_* is flat band potential [28]. In the function of 1/*C*^2^ and applied potential (*V*), $V_{fb}+\frac{k_{B}T}{e}$ can be determined by taking the x-intercept of a linear fit to the Mott–Schottky plot (shown in Figure 6b), and $\frac{k_{B}T}{e}$ was calculated as 0.0259 V. Thus, the *V_fb_* of BFO is estimated as −0.33 V (vs. Ag/AgCl) or 0.27 V (vs. reversible hydrogen electrode, RHE), and BFMO is −0.41 V (vs. Ag/AgCl) or 0.20 V (vs. RHE). To convert the obtained potential (vs. Ag/AgCl) to RHE, the following equation [29] was used:

*E*(RHE) = *E*(Ag/AgCl) + 0.059 pH + 0.21 (3)

For n-type semiconductors, the flat-band potential (*V_fb_*) was considered to be located just under the CB; hence, we can calculate the band position as shown in Figure 6e [6]. Even though the band positions obtained by the three methods were found to vary, one fact is that they can all be classified into the structure shown in Figure 6f, in which the band bends and benefits the separation of photogenerated e–h.

**Figure 7:**
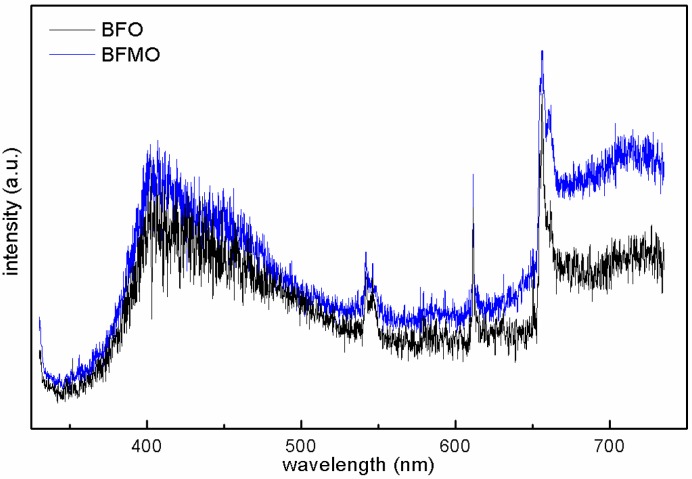
Photo-luminescence spectra of BFO and BFMO films.

Although BFMO has a smaller band gap than BFO, and BFO/BFMO films own an advantageous band bend causing an internal field, pure BFO film performs best compared with BFMO and the heterostructured BFO/BFMO films. These facts imply that another factor influencing the photo response, which exceeds the effect of more light absorption, exists in the BFMO layers and the band bend in the interface of BFO/BFMO. On the basis of the XRD patterns (Figure 1), the inferior crystallinity of BFMO implied more vacancy defects in the BFMO films. More vacancy defects in BFMO can also be determined in photo-luminescence (PL) spectra (Figure 7). The fluorescence effect is a little bit higher in BFMO than in BFO films, which might be attributed to the higher e–h combination occurring around the defects. The doping process could generate more defects in BFMO, and the consequent higher e–h combination considerably decreased the PEC properties of BFMO and BFO/BFMO films. Nevertheless, Mn single doping does not cause detrimental effects on photo response of all material systems; for example, Mn single doping into the TiO_2_ lattice results in a significant enhancement in photoabsorption and in the quantity of photogenerated hydroxyl radicals due to the formation of a low oxygen-vacancy content [30].

## 3. Materials and Methods

The BFO films were fabricated on FTO substrates as reported previously [10]. To get the 5% Mn-doped BFO, we chose C_4_H_6_MnO_4_·4H_2_O as the Mn source. The BFO film is 4-layered, the BFMO film is 4-layered, and the heterostructured BFO/BFMO film (shown in Figure 2c) is combined by 2-layered BFO and 2-layered BFMO. One layer represents one instance of spin coating.

XRD was carried out to analyze the crystal structure by X-ray diffractometer (RIGAKU D/max 2500, Tokyo, Japan) with Cu radiation at 40 kV/200 mA. The XRD scanning speed was 6°/min (standard configuration). The microstructures were checked by field emission microscope at 20 kV (JSM-7001F, JEOL, Tokyo, Japan). UV-Vis absorption spectra were measured by a UV-Vis spectrophotometer (UV-3310, HITACHI, Tokyo, Japan). The PL spectra were tested with LabRAM HR Evolution (HORIBA, Kyoto, Japan), and the incident laser wave length was 325 nm. XPS valance spectrum was tested by PHI Quantera SXM (ULVAC-PHI, INC., Tokyo, Japan).

The PEC measurements, including the time dependence open circuit potential (*P*-*t*), the time dependence of the photocurrent density (*I*-*t*) was carried out by an electrochemistr workstation (CHI 660D, CHENHUA, Shanghai, China) in a three-electrode cell. The prepared samples, a small Pt foil, and a saturated Ag/AgCl were used as the working electrode, the counter electrode, and the reference electrode, respectively. 0.1 M Na_2_SO_4_ (at pH = 6.7) was used as the electrolyte. The working electrode was illuminated under 150 mW/cm^2^ visible-light irradiation (λ>420 nm). *I*-*t* was measured in the light on–off process with a pulse of 10–50 s by the potentiostatic technique at 0 V, −0.1 V, 0.1 V and −0.4 V bias versus Ag/AgCl.

## 4. Conclusions

In this work, we carried out band engineering on BFO films via Mn doping and prepared heterostructured BFO/BFMO films. The photo-response activities of these films were studied through PEC tests. The pure BFO film is sensitive to visible light, which is potentially applicable in photo-related fields, such as optical sensitive sensors, PV cells, and photocatalysts. In our heterostructured BFO/BFMO samples, the facilitating effects provided by the smaller band gap of the BFMO layer and the internal field in the interface on PEC performance cannot compensate the negative effect caused by the severe e–h combination in the BFMO layer. The combination of photogenerated charge carriers occurred around the defects introduced by Mn doping. The major results indicated that, to enhance the photo-response ability and improve energy conversion when designing a BFO based photic device, integrated multi-factors need to be taken into account. In addition, more theoretical work with respect to the separation mechanism of photogenerated charge carriers is desirable in the future.
